# The sigma‐1 receptor behaves as an atypical auxiliary subunit to modulate the functional characteristics of Kv1.2 channels expressed in HEK293 cells

**DOI:** 10.14814/phy2.14147

**Published:** 2019-06-20

**Authors:** Madelyn J. Abraham, Kayla L. Fleming, Sophie Raymond, Adrian Y. C. Wong, Richard Bergeron

**Affiliations:** ^1^ Department of Cellular and Molecular Medicine University of Ottawa Ottawa Ontario Canada; ^2^ Neuroscience Ottawa Hospital Research Institute Ottawa Ontario Canada

**Keywords:** apFRET, ion channel biophysics, Kv*β* subunit, motor neuron disease, voltage‐clamp electrophysiology

## Abstract

Expression of Kv1.2 within Kv1.x potassium channel complexes is critical in maintaining appropriate neuronal excitability and determining the threshold for action potential firing. This is attributed to the interaction of Kv1.2 with a hitherto unidentified protein that confers bimodal channel activation gating, allowing neurons to adapt to repetitive trains of stimulation and protecting against hyperexcitability. One potential protein candidate is the sigma‐1 receptor (Sig‐1R), which regulates other members of the Kv1.x channel family; however, the biophysical nature of the interaction between Sig‐1R and Kv1.2 has not been elucidated. We hypothesized that Sig‐1R may regulate Kv1.2 and may further act as the unidentified modulator of Kv1.2 activation. In transiently transfected HEK293 cells, we found that ligand activation of the Sig‐1R modulates Kv1.2 current amplitude. More importantly, Sig‐1R interacts with Kv1.2 in baseline conditions to influence bimodal activation gating. These effects are abolished in the presence of the auxiliary subunit Kv*β*2 and when the Sig‐1R mutation underlying ALS16 (Sig‐1R‐E102Q), is expressed. These data suggest that Kv*β*2 occludes the interaction of Sig‐1R with Kv1.2, and that E102 may be a residue critical for Sig‐1R modulation of Kv1.2. The results of this investigation describe an important new role for Sig‐1R in the regulation of neuronal excitability and introduce a novel mechanism of pathophysiology in Sig‐1R dysfunction.

## Introduction

Delayed rectifier voltage‐gated potassium channels play an essential role in determining the threshold for action potential firing and subsequent neuronal repolarization (Sutherland et al. 1999). Among the *Shaker‐*type Kv1.x family, Kv1.2 is especially important in allowing neurons to adapt to repetitive trains of depolarization via a unique regulatory mechanism termed “use‐dependent activation” (Baronas et al. 2015, 2016) or “prepulse potentiation” (Grissmer et al. 1994). Although Kv1.2 channels are unique in their ability to generate use‐dependent activation, they will confer this property to Kv1.2‐containing Kv1.x channel heteromers (Baronas et al. 2015). Thus, the presence of Kv1.2 subunits increases the threshold for neuronal firing (Brew et al. 2007) and terminates bursts of action potentials (Palani et al. 2010), thereby protecting cells from hyperexcitability.

Use dependence is defined as the ability of Kv1.2 to display bimodal activation gating, with channels able to transition to a single open state along either “fast” or “slow” activation pathways (Rezazadeh et al. 2007). Upon depolarization, binding affinity for a hitherto unidentified extrinsic regulator is reduced, causing disinhibition of channel activation and allowing Kv1.2 to open along the “fast” activation pathway (Baronas et al. 2016). Although this extrinsic gating regulator has yet to be identified, there is strong evidence that it is a protein that interacts with the Kv1.2 at the S2‐S3 linker in the closed conformation (Rezazadeh et al. 2007).

The Sig‐1R is an endoplasmic reticulum (ER)‐resident protein that translocates to other cellular compartments, including the plasma membrane, upon ligand activation (Su et al. 2010; Kourrich 2017), and modulates a plethora of potassium channels (Soriani et al. 1999; Wilke et al. 1999; Lupardus et al. 2000; Aydar et al. 2002; Zhang and Cuevas 2005; Martina et al. 2007; Kinoshita et al. 2012; Wong et al. 2016). Sig‐1R seems to meet all the criteria as an auxiliary subunit for Kv1.x channels, as it can modulate Kv1.x biophysical properties (Aydar et al. 2002; Kinoshita et al. 2012), and facilitates Kv1.x trafficking to the plasma membrane (Kourrich et al. 2013; Delint‐Ramirez et al. 2018). Previous work has examined the effect of Sig‐1R ligand activation on the biophysical properties of Kv1.3, Kv1.4, and Kv1.5 (Aydar et al. 2002; Kinoshita et al. 2012); however, studies of Sig‐1R interactions with Kv1.2 channels are notably absent.

In this study, we addressed several questions related to Sig‐1R modulation of Kv1.2 channels. Firstly, we investigated how ligand activation of Sig‐1R modulates Kv1.2 biophysical properties. Secondly, we determined whether expression of the Kv1.x‐specific auxiliary subunit, Kv*β*2, may occlude any regulatory interaction of Sig‐1R to Kv1.2. Lastly, we examined whether the regulatory interaction between Kv1.2 and Sig‐1R is altered following expression of the Sig‐1R mutation underlying ALS16, Sig‐1R‐E102Q (Al‐Saif et al. 2011). In our results, we show a direct interaction between the Sig‐1R and Kv1.2 under control conditions and that ligand activation of the Sig‐1R inhibits Kv1.2 channel current. Moreover, simply increasing expression levels of the Sig‐1R leads to a change in the activation gating state of Kv1.2, from predominantly “fast” to predominantly “slow.” These effects are not observed in the presence of Sig‐1R‐E102Q, suggesting that aberrant Kv1.2 channel modulation may underlie neuronal hyperexcitability observed in ALS (Do‐Ha et al. 2018; Fogarty 2018).

## Materials and Methods

### Cell culture and cDNA transfection

All experiments were performed on HEK293 cells grown in Dulbecco's modified Eagle medium (DMEM: Wisent Bioproducts, Montreal, QC, Canada), containing 10% fetal bovine serum (FBS), 100 U/mL penicillin/streptomycin, and 1X GlutaMAX (Thermo Fisher Scientific, Waltham, MA) in a humidified 37°C, 5% CO_2_ incubator. Cells were washed with phosphate buffered saline (PBS: 10 mmol/L Na_2_HPO_4_, 1.76 mmol/L KH_2_PO_4_, 137 mmol/L NaCl, and 2.68 mmol/L KCl; pH 7.2) and passaged upon reaching 80% confluence (roughly every 3–4 days) using trypsination (0.05% trypsin; Thermo Fisher Scientific). For live‐cell imaging and electrophysiology experiments, cells were plated on either 35‐mm *μ*‐Dishes (ibidi GmbH, Martinsreid, Germany) or 15‐mm Thermanox (Thermo Fisher Scientific) plastic coverslips at a density of 2 × 10^6^ cells/mL. The day following plating, cells were transiently transfected using TransIT‐2020 transfection reagent (Mirus Bio, Madison, WI) as per manufacturer protocol. Imaging or electrophysiology experiments were performed 24–48 h post‐transfection.

### cDNA constructs

The Sig‐1R‐YFP, Sig‐1R‐E102Q‐YFP, and Sig‐1R‐mCh constructs used in this study were generated as previously described (Wong et al. 2016). To generate the mutant Sig‐1R‐E102Q‐mCh construct, the Sig‐1R‐E102Q gene was subcloned from Sig‐1R‐E102Q‐YFP into a viral pLVX‐Ef1a‐mCh backbone (a kind gift from Ruth Slack, University of Ottawa, Ottawa, ON, Canada) using EcoRI and BamHI restriction sites. Prior to ligation, the digested mutant Sig‐1R‐E102Q gene fragment was gel‐isolated and purified using EtOH‐NaCl DNA precipitation. Following ligation, the plasmid was then used to transform chemically competent NEB Turbo *E*. *coli* (New England BioLabs, Ipswich, MA). Positive clones were then screened using sequencing primers:

Sig‐1R FWD: 5′‐GCTGCAAGTGGGTATTTGTGA‐3′

Sig‐1R RV: 5′‐ACTTTTCGTGGTGCCCTCTT‐3′

The cDNA constructs for Kv1.2, Kv1.2‐GFP, Kv1.5, and Kv*β*2 were obtained from Origene (Origene Global, Rockville, MD). All constructs were expressed on a pCMV6 vector containing either kanamycin/neomycin or ampicillin resistance.

### Drugs and solutions

All electrophysiological experiments were performed with cells in an external bath solution containing (mmol/L): 150 NaCl, 10 HEPES, 3 KCl, 1 MgSO_4_, 2 CaCl_2_, and 1 Na‐ascorbate, adjusted to pH 7.4 with 5N NaOH. In experiments where the KCl concentration was increased to 135 mmol/L, the concentration of NaCl was reduced to 19 mmol/L to maintain the total monovalent ion concentration at 154 mmol/L. Thick‐walled borosilicate glass electrodes (1.5 mm OD, 0.9 mm ID; Sutter Instruments, Novato, CA) were filled with an internal K+ gluconate solution containing (mmol/L): 115 K^+^‐Glu, 20 KCl, 10 HEPES, 4 Mg^2+^‐ATP, 0.5 Na^+^‐GTP, and 10 mmol/L Na^+^‐phosphocreatine. Internal solution pH was adjusted to 7.4 using 5N KOH. Osmolarity of both solutions was adjusted to 290 mOsm using sucrose. E_k_ was calculated to be −80 mV using the Nernst equation. All salts were obtained from Sigma‐Aldrich, Canada (Oakville, ON, Canada).

All drugs were bath applied to cells in a continuous flow bath. Bath volume was ~1 mL, with the flow rate set to ~1 mL/min for all experiments. The Sig‐1R agonists (2S,6S,11S)‐1,2,3,4,5,6‐Hexahydro‐6,11‐dimethyl‐3‐(2‐propenyl)‐2,6‐methano‐3‐benzazocin‐8‐ol hydrochloride (SKF 10,047; SKF) and 2‐(4‐Morpholinethyl) 1‐phenylcyclohexanecarboxylate hydrochloride (PRE‐084; PRE) were obtained from Tocris (Ellisville, MO). SKF was dissolved in ddH_2_O to a stock concentration of 50 mmol/L and was added to cell bath solutions to achieve a final concentration of 50 *μ*mol/L. Similarly, PRE‐084 was dissolved in ddH_2_O to a stock concentration of 10 mmol/L, and was added to cell bath solutions to achieve a final concentration of 10 *μ*mol/L. In this study, the term “agonist” refers to a Sig‐1R ligand which can induce a Sig‐1R‐mediated physiological effect.

### Voltage‐clamp electrophysiology

Whole‐cell voltage‐clamp recordings were performed on HEK293 cells transiently expressing Kv1.2 and eYFP, Sig‐1R‐YFP, Sig‐1R‐E102Q, or Kv*β*2 together with either eYFP or Sig‐1R‐YFP. Cells were imaged under epifluorescence, and only cells displaying YFP fluorescence were selected for recordings. HEKs were held at a membrane potential of −60 mV, and for pharmacological experiments, 1‐sec depolarizations to +20 mV every 40 sec were given, following a 500‐msec hyperpolarizing step to −80 mV to ensure full recovery from inactivation. This elicited stable Kv1.2‐mediated whole‐cell currents for the entire duration of the experiment, typically 50–60 min (but up to 90 min) with little current rundown. All other experiments were carried out using a 1‐sec depolarization and a 40‐sec intersweep interval unless otherwise stated. Data were acquired and analyzed using the pClamp 10.4 suite (Molecular Devices, San Jose, CA) with a sampling rate of 10 kHz and a low‐pass Bessel filter set at 2 kHz. All traces were post hoc leak‐subtracted prior to analysis using a 1/10 step protocol as previously described (Wong et al. 2016).

Current‐voltage (IV) plots were generated by normalization of peak amplitudes elicited by 1‐sec depolarizations from −80 to +80 mV in 20 mV increments unless otherwise stated. Voltage dependence of inactivation plots was generated by expressing peak current at +80 mV relative to the current amplitude at the end of a 5 sec prepulse. Voltage dependence of activation plots was generated by normalization of channel conductance (G) to *G*_max_ at +60 mV, which was best fit with a single Boltzmann function to derive *V*_1/2_, using the formula:

$$Y=\frac{\text{Min}+(\text{Max}-\text{Min})}{1+\exp\left(\frac{V_{1/2}-V_{m}}{k}\right)}$$

where *V*_1/2_ is the voltage at which *Y* = 0.5, *V*_m_ is the membrane voltage, and *k* is the slope factor, in mV.

Channel activation kinetics were best described with either a double or a single exponential function. To facilitate comparison, the double exponential function was converted to a weighted exponential using the formula:

$$\tau \left(\text{weighted}\right)=A1\times \exp\left(\frac{-t}{\tau 1}\right)+A2\times \exp\left(\frac{-t}{\tau 2}\right)$$

where *A*_1_ and *τ*_1_ are the area and decay time constant of the first exponential and *A*_2_ and *τ*_2_ are the area and decay time constant of the second exponential function.

### FRET microscopy

FRET experiments were performed on HEK293 cells expressing equimolar amounts of Sig‐1R‐mCh and Kv1.2‐GFP using a Zeiss LSM880 AxioObserverZ1 Confocal Microscope (Zeiss GmbH, Oberkochen, Germany), with excitation wavelengths of 561 nm and 488 nm for mCh and GFP, respectively. Cells were imaged in Phenol Red free MEM (Wisent Bioproducts) containing 10% FBS on a prewarmed 37°C stage with 5% CO_2_, using a 63× (NA 1.4) oil immersion objective (Zeiss). A resolution of 512 × 512 pixels was used, with a dwell time of 0.5 *μ*sec/pixel. Following five frames of prebleach baseline, a square region of interest (ROI) was bleached at 80% 561 nm laser power for 2.5 sec. 15 frames were captured postbleach. FRET efficiency was measured via increased donor (GFP) emission following acceptor photobleaching (mCh), using the formula:

$$E=1-(I_{\mathrm{pre}}/I_{\mathrm{post}})$$

where *I*_pre_ and *I*_post_ are the fluorescent intensities of the donor before and after photobleaching (Bajar et al. 2016). Mean fluorescent intensities within the ROI were determined using Fiji (Schindelin et al. 2012) and were background subtracted prior to analysis. As an internal control, FRET efficiency was calculated within a nonbleached ROI of each cell to account for false‐positive FRET signals.

### Western blotting

HEK293 cells were transfected with Kv*β*2 or Sig‐1R‐YFP as previously described. Twenty 4 h following transfection, cells were lysed on ice with 500 *μ*L radioimmunoprecipitation buffer (RIPA: 150 mmol/L NaCl, 50 mmol/L Tris, 0.5% sodium deoxycholate, 0.1% NP‐40, 5 mmol/L sodium pyrophosphate, 2 mmol/L *β*‐glycerophosphate, 1 × EDTA‐free protease inhibitor (Fisher Scientific Canada, Nepean, ON, Canada)), at pH 7.5, and total protein concentration was determined with DC protein assay (Bio‐Rad Laboratories Canada, Mississauga, ON, Canada). In cases where cells were treated with 50 *μ*mol/L SKF, treatments were applied 24 h post‐transfection and collected after 20 min. Total protein (1.5 mg per lane) was resolved on Tris‐glycine SDS‐PAGE and transferred onto PVDF membranes and probed using a Rabbit‐polyclonal anti‐KCNAB2 (1:500; OriGene Technologies, Rockville, MD), anti‐Sigmar1 (1:500; Atlas Antibodies, Stockholm, Sweden), and HRP‐conjugated rabbit secondary antibody (1:15000; Jackson ImmunoResearch, West Drove, PA).

After incubation with primary and secondary antibodies dissolved in 5% milk, membranes were developed using Luminata Forte (MilliporeSigma, Burlington, MA) and visualized using LI‐COR Odyssey Fc (LI‐COR, Lincoln, NE). Band intensities were normalized to total protein as determined by Fast Green stain (125 *μ*mol/L Fast Green FCF (Sigma‐Aldrich), 6.7% acetic acid, 30% methanol), or by Ponseau S stain (0.1% Ponceau S (w/v: Sigma‐Aldrich), 5% acetic acid). This was done to ensure that normalization did not rely on a single protein, but rather the total profile of protein found in the crude cellular extract (Li and Shen 2013).

### Analysis and statistics

Origin 8.5 (OriginLab, Northampton, MA) was used for graph design, statistics, and curve fitting. Unless otherwise stated, all data are presented as mean ± 95% CI in bar and symbol/line plots where error bars are shown. Each point in a scatterplot represents the data from a single cell. In box and whisker plots, boxes show the median, as well as first and third quartile, with the mean shown as a filled symbol. Whiskers represent Tukey's fences, defined as 1.5× above and below the interquartile range. Data points outside these ranges were treated as statistical outliers and removed. “*n*” numbers reported for electrophysiological experiments represent individual cells. Pharmacological experiments were performed on only one cell per transfected coverslip from at least three separate transfections over multiple experimental days. For FRET experiments, “*n*” represents the number of individual cells. In these experiments, multiple cells were imaged per dish, from three separate transfections over multiple experimental days.

Post hoc power analysis reveals that all comparisons are adequately powered at a level >80%, assuming an *α* = 0.05. Statistical significance was determined using ANOVA for multiple comparisons or comparison of independent groups, while a paired Student's *t*‐test was used for pharmacological comparisons. Statistical significance was achieved if *P* < 0.05. Unless otherwise stated, single asterisks (*) represent *P* < 0.05, while double asterisks (**) indicate *P* < 0.005.

## Results

### Ligand activation of Sig‐1R decreases Kv1.2 current amplitude

Increasing evidence from electrophysiological studies has shown that Sig‐1R modulation of Kv1.x channels is subtype‐dependent. Pharmacological activation of the Sig‐1R decreases Kv1.4 and Kv1.5 current amplitude and accelerates Kv1.4 inactivation kinetics in recombinant *Xenopus* oocytes (Aydar et al. 2002). In contrast, Kv1.3 current amplitude is not affected by treatment with Sig‐1R ligands (Kinoshita et al. 2012). It is known that Sig‐1R activation by cocaine (Sharkey et al. 1988) promotes trafficking of Kv1.2 to the plasma membrane (Kourrich et al. 2013; Delint‐Ramirez et al. 2018), but it is unknown whether Sig‐1R modulates the biophysical properties of Kv1.2. Analogous to what is observed in other Kv1.x channels, we hypothesized that activation of the Sig‐1R may affect Kv1.2 current amplitude and voltage dependence of inactivation.

**Figure 1:**
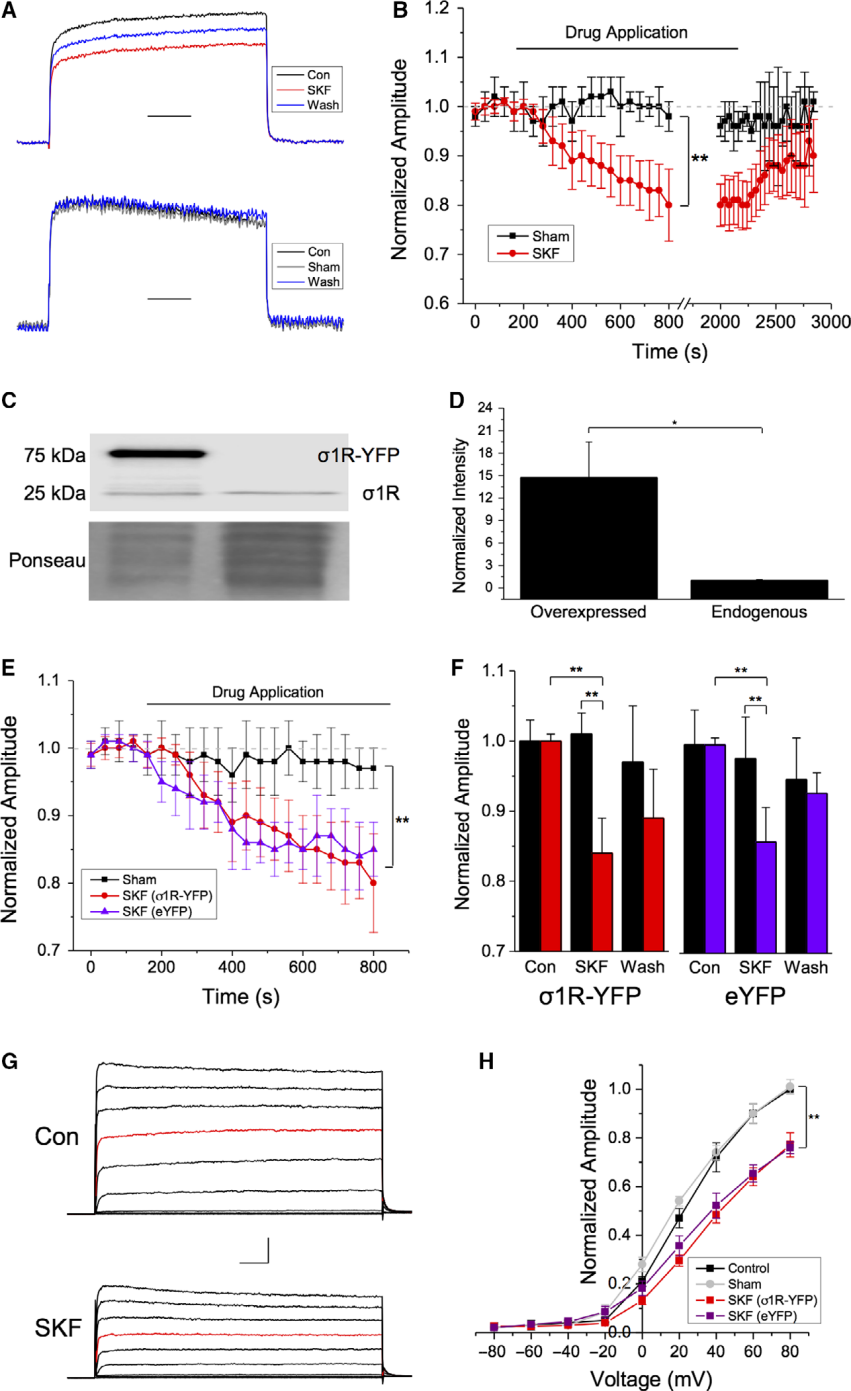
Bath application of the Sig‐1R agonist SKF‐10,047 decreases Kv1.2 current amplitude. (A–B) Representative voltage‐clamp traces showing normalized Kv1.2 channel current evoked by a 1 sec depolarizing step from −80 to +20 mV in the absence (black) and presence (red) of SKF 10,047 (SKF) and following SKF washout (blue). Scale bar is 200 msec. Sham drug applications had no effect on current amplitude, which showed no significant rundown over the course of the experiment (B, black). Administration of SKF resulted in a ~30% decrease in current amplitude (B, red) which was partially reversible upon washout. (C) Representative Western blot showing the protein level of Sig‐1R in HEK293 cell lysates following transient transfection of Sig‐1R‐YFP compared to endogenous Sig‐1R. (D) Densiometric quantification and normalization of the band intensities to a Ponseau stain revealed a ~40 × increase in Sig‐1R levels when Sig‐1R‐YFP was overexpressed. (E–F) Overexpression of the Sig‐1R had no additional effect on the decrease in Kv1.2 current amplitude observed in the presence of SKF (E, red vs. purple traces; F, red vs. purple bars). (G) Representative traces from a current‐voltage (IV) plot obtained in control (top) and SKF conditions (bottom), from −80 to +80 mV in 20 mV increments. The step to +20 mV is shown in red for clarity. (H) There was a significant decrease in current amplitude at all voltages greater than 0 mV in the presence of SKF in cells overexpressing Sig‐1R (H, red trace) and in cells with endogenous levels of Sig‐1‐R expression (H, purple trace). The IV response to sham cells was not significantly different from control (H, gray trace). Scale bar is 100 msec and 200 pA. Data are expressed as mean ± 95% CI. Asterisks indicate statistical significance; single asterisks (*) represent *P* < 0.05, while double asterisks (**) indicate *P* < 0.005.

We used voltage‐clamp electrophysiology in HEK293 cells transiently transfected with Kv1.2 and Sig‐1R‐YFP (1:1 cDNA ratio by mass) to characterize the effect of Sig‐1R ligand‐activation on Kv1.2 channels. Figure 1A shows typical responses of Kv1.2 channels following a 1‐sec depolarization from −80 to +20 mV, given every 40 sec. Bath application of the selective Sig‐1R agonist SKF 10,047 (Zukin et al. 1986) resulted in a statistically significant decrease in Kv1.2 current amplitude (20.1 ± 7.3%; Student's paired *t*‐test, *P* = 0.001; *n* = 15) which was maintained for the duration of agonist application (up to 30 min). Following washout of SKF, a partial recovery to ~90% of control amplitude was observed (Fig. 1A, top, blue trace; Fig. 1B, red trace). This was not observed when a sham experiment was performed. Here, the current remained consistent for the entire duration of the experiment, up to 50 min (Fig. 1A, bottom; Fig. 1B, black trace; *n* = 6).

As expression of the Sig‐1R alone is sufficient to modulate Kv1.x channel function (Aydar et al. 2002; Kinoshita et al. 2012), we were concerned that effect of SKF that we observed was simply due to the ~7× higher expression level of Sig‐1R‐YFP following transient transfection compared with endogenous Sig1‐R levels (Fig. 1C and D; *P* = 0.001; *n* = 4). Therefore, we repeated the experiment in HEK293 cells that had been transiently transfected with Kv1.2 and enhanced YFP (eYFP). Administration of SKF to these cells resulted in a 17 ± 4.2% decrease in Kv1.2 channel amplitude (Fig. 1E; purple trace), very similar to that observed in cells transfected with Kv1.2 and Sig‐1R‐YFP (Fig. 1F; red bars). Thus, we demonstrate that increasing the level of Sig‐1R expression has no additional effect on channel amplitude in response to SKF administration.

We next tested the voltage dependence of this SKF‐induced decrease in Kv1.2 channel amplitude by performing 1‐sec depolarizations from −80 mV to membrane potentials between −80 and +80 mV in +20 mV increments to generate a current–voltage (IV) plot (Fig. 1G). As expected, sham drug application had no significant effect on current amplitude (Fig. 1H; gray line; *P* = 0.97; *n* = 6). However, bath application of SKF led to a significant decrease in channel amplitude (*P* < 0.005; Student's paired *t*‐test) at all membrane potentials greater than +40 mV in cells transfected with Kv1.2 and Sig‐1R‐YFP, with a 28.2 ± 3.1% decrease in current amplitude at +80 mV (Fig. 1H; red line; *P* = 0.003; *n* = 10). Similar data were obtained from cells transfected with Kv1.2 and eYFP, where a 22.2 ± 2.9% decrease in current amplitude at +80 mV was observed upon application of SKF (Fig. 1H, purple line; *P* = 0.007; *n* = 8). Comparison of both SKF treated groups showed no significant difference between them (*P* = 0.85; *n* = ~8–10).

**Figure 2:**
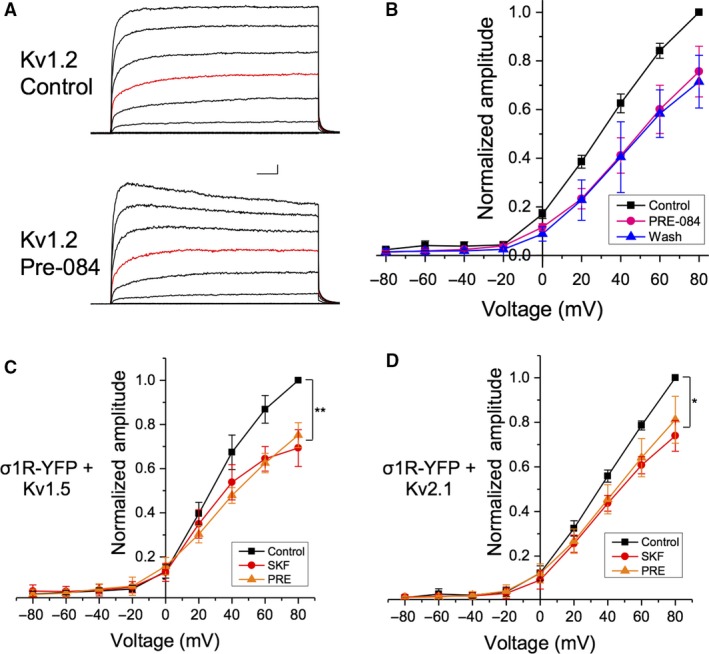
Sig‐1R pharmacological activation with SKF and PRE‐084 causes a decrease Kv channel current amplitude. (A) Representative IV plots from cells co‐transfected with Sig‐1R‐YFP and Kv1.2 before (top) and during (bottom) bath application of 10 *μ*mol/L PRE‐084 (PRE), with the +20 steps shown in red. (B) There was a significant decrease in channel amplitude in the presence of PRE (B, pink) which was not reversed upon agonist washout (B, blue). Scale bar is 200 msec and 200 pA. (C–D) Bath application of either SKF (red) or PRE (orange) had similar effects on current amplitude in cells co‐transfected with Sig‐1R‐YFP and either Kv1.5 (C) or Kv2.1 (D). Data are expressed as mean ± 95% CI. Asterisks indicate statistical significance; single asterisks (*) represent *P* < 0.05, while double asterisks (**) indicate *P* < 0.005.

To ensure that the observed effect of SKF was due to Sig‐1R activation, we repeated our IV experiments using another selective Sig‐1R agonist, PRE‐084 (10 *μ*mol/L), in cells co‐transfected with Kv1.2 and Sig‐1R‐YFP (Fig. 2A). Bath administration of PRE‐084 also resulted in a ~20% decrease in Kv1.2 current amplitude at all membrane potentials more depolarized than +40 mV (Fig. 2B, orange; *P* = 0.003; *n* = 8). In contrast to SKF, we observed no recovery following washout of PRE‐084 (Fig. 2B, blue). We also administered both SKF and PRE to HEK293 cells transfected with Sig‐1R‐YFP and Kv1.5 (Fig. 2C) or Sig‐1R‐YFP and Kv2.1 (Fig. 2D) to rule‐out any Kv1.2‐specific effects of these Sig‐1R ligands. There was a significant decrease in Kv1.5 current amplitude following bath administration of SKF (31 ± 6.1%; Fig. 2C, red; *P* = 0.004; *n* = 6) in agreement with previously published data (Aydar et al. 2002). We further demonstrate that PRE‐084 has a comparable effect to SKF, resulting in a ~40% decrease in Kv1.5 current amplitude (Fig. 2C, orange; *n* = 5). When the Sig‐1R agonists were added to cells transfected with Sig‐1R‐YFP and Kv2.1, bath application of PRE‐084 decreased Kv2.1 currents by 19 ± 8.2% (Fig. 2D, orange; *P* = 0.037; *n* = 5) also in agreement with recent work (Liu et al. 2017). Bath application of SKF resulted in a ~25% decrease in current amplitude (Fig. 5D; red; *P* = 0.022; *n* = 4) of Kv2.1, which was not significantly different to that observed following PRE‐084 application (*P* = 0.79).

Taken together, our data show that administration of Sig‐1R agonists leads to a small, but significant decrease in Kv1.2 channel amplitude, which is likely due to Sig‐1R activation.

### Sig‐1R directly interacts with Kv1.2

Previous results from co‐immunoprecipitation (co‐IP) studies provide indirect evidence of a direct protein‐protein interaction between the Sig‐1R and *Shaker*‐type voltage‐gated potassium channels (Kv1.x channels) and demonstrate that Sig‐1R agonist application facilitates an increase in interaction between these proteins (Kourrich et al. 2013; Delint‐Ramirez et al. 2018). Results suggest that the Sig‐1R interacts with Kv1.3 and Kv1.4 in *Xenopus* recombinant systems (Aydar et al. 2002; Kinoshita et al. 2012) and with Kv1.2 in mouse prefrontal cortex and nucleus accumbens (Kourrich et al. 2013). Thus, we speculated that the modulation of Kv1.2 channel amplitude following administration of SKF to HEK293 cells may also be accompanied by a change in interaction between Kv1.2 and Sig‐1R.

**Figure 3:**
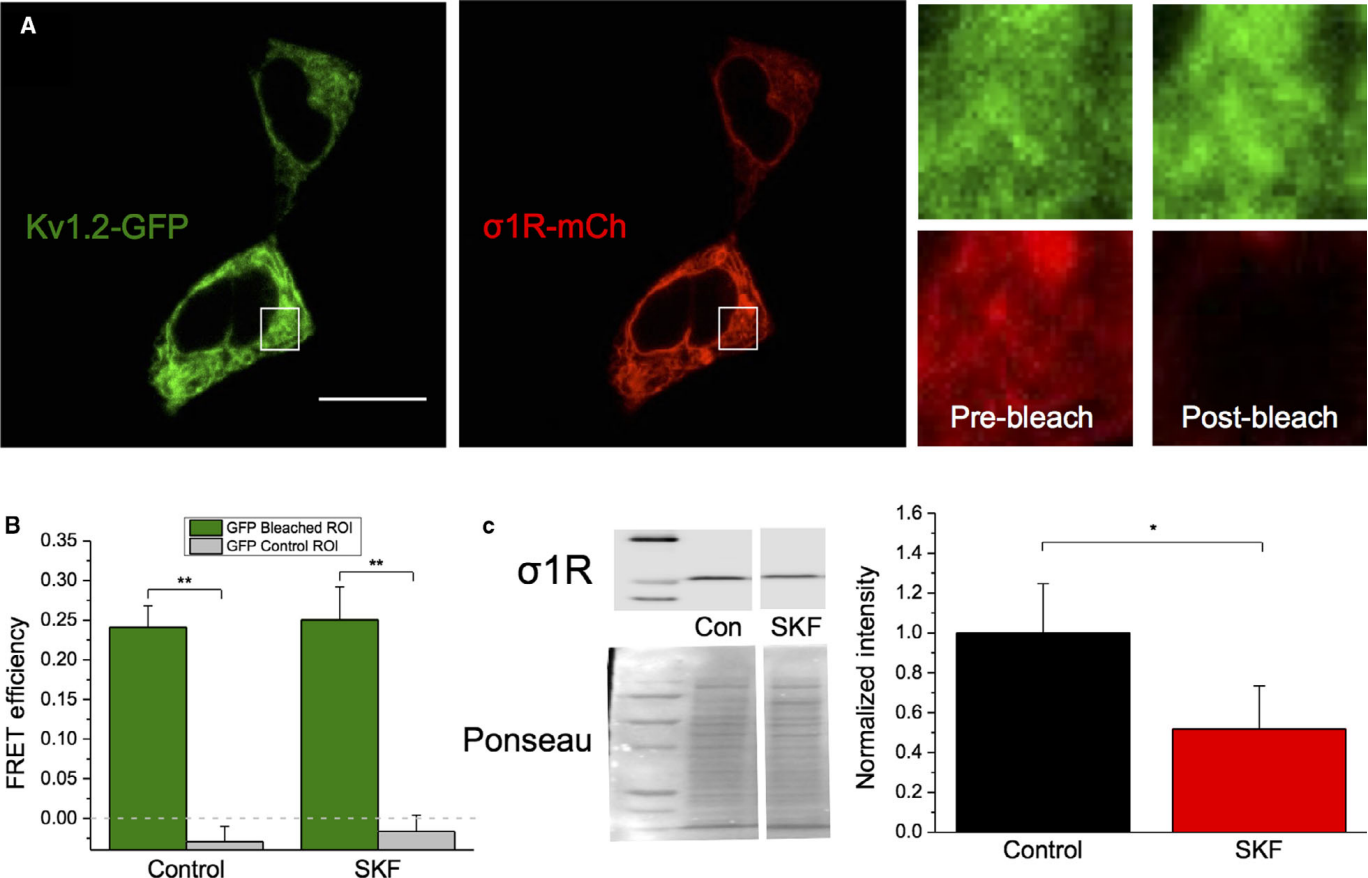
No change in interaction between Kv1.2 and Sig‐1R following SKF application. (A) Representative confocal images of HEK293 cells transiently co‐expressing Kv1.2‐GFP and Sig‐1R‐mCh (σ1R‐mCh; *A*_i_). Magnification of the square ROI displays Kv1.2‐GFP and Sig‐1R‐mCh fluorescence before and after acceptor photobleaching (*A*_ii_). Scale bar in Panel A represents 10 *μ*m. (B) There was a significant increase of FRET efficiency in the bleached ROI versus the control nonbleached ROI in all groups. No significant differences in FRET efficiency between untreated and treated cells were observed after 20‐min SKF. (C) Western blot experiments show that there is a significant decrease in total Sig‐1R protein level following 20‐min SKF application. Data are expressed as mean ± 95% CI. Asterisks indicate statistical significance; single asterisks (*) represent *P* < 0.05, while double asterisks (**) indicate *P* < 0.005.

To this end, we performed acceptor photobleaching Fluorescence Resonance Energy Transfer (apFRET) in HEK293 cells co‐transfected with Kv1.2‐GFP (the donor fluorophore) and Sig‐1R‐mCherry (mCh; the acceptor fluorophore) in the presence and absence of SKF. apFRET is dependent on emission energy transfer from the co‐expressed fluorescent donor to the acceptor, such that excitation of the acceptor will quench donor emission when the proteins are in close proximity (Bajar et al. 2016; Martin et al. 2018). FRET efficiency calculations were performed by measuring mean GFP intensity per frame before (prebleach panels in Fig. 3A) and after mCh bleaching (postbleach panels in Fig. 3A). A control, nonbleached ROI was used to control for false‐positive FRET efficiencies in each cell (Organ‐Darling et al. 2013).

Prior to SKF treatment, FRET efficiency was found to be 21.8 ± 1.8% in the bleached ROI, versus 0.82 ± 1.6% in the control region (Fig. 3B, *P* = 5.8 × 10^−12^, one‐way ANOVA; *n* = 29). These results further support the finding that Kv1.2 and Sig‐1R are interacting in baseline conditions (Kourrich et al. 2013). When HEK293 cells were treated with SKF for 20 min (to replicate the time course of electrophysiological experiments), there was no significant change in FRET efficiency compared with the control cells (Fig. 3B; *P* = 0.99; *n* = 21). Intriguingly, we observed a significant decrease in Sig‐1R total protein levels in the presence of SKF (Fig. 3C; *P* = 0.03; *n* = 7). Therefore, we clearly demonstrate that the Sig‐1R directly interacts with Kv1.2 in the absence of ligand and that this interaction is unchanged following SKF application, despite a decrease in the overall level of Sig‐1R protein expression.

Taken together, these results thus far demonstrate that acute pharmacological activation of the Sig‐1R by SKF acutely modulates Kv1.2 channels. These effects are likely due to ligand‐dependent changes in Sig‐1R activity, as opposed to changes in Sig‐1R recruitment to Kv1.2 channels.

### Pharmacological activation of Sig‐1R has no effect on the voltage dependence of Kv1.2 inactivation

Sig‐1R activation by SKF has been shown to accelerate inactivation kinetics of Kv1.4 channels (Aydar et al. 2002), while overexpression of Sig‐1R has the same effect on Kv1.3 expressed in *Xenopus* oocytes (Kinoshita et al. 2012). Thus, we speculated that administration of SKF would have an effect on the inactivation profile of Kv1.2 and that this may be exacerbated with Sig‐1R overexpression.

**Figure 4:**
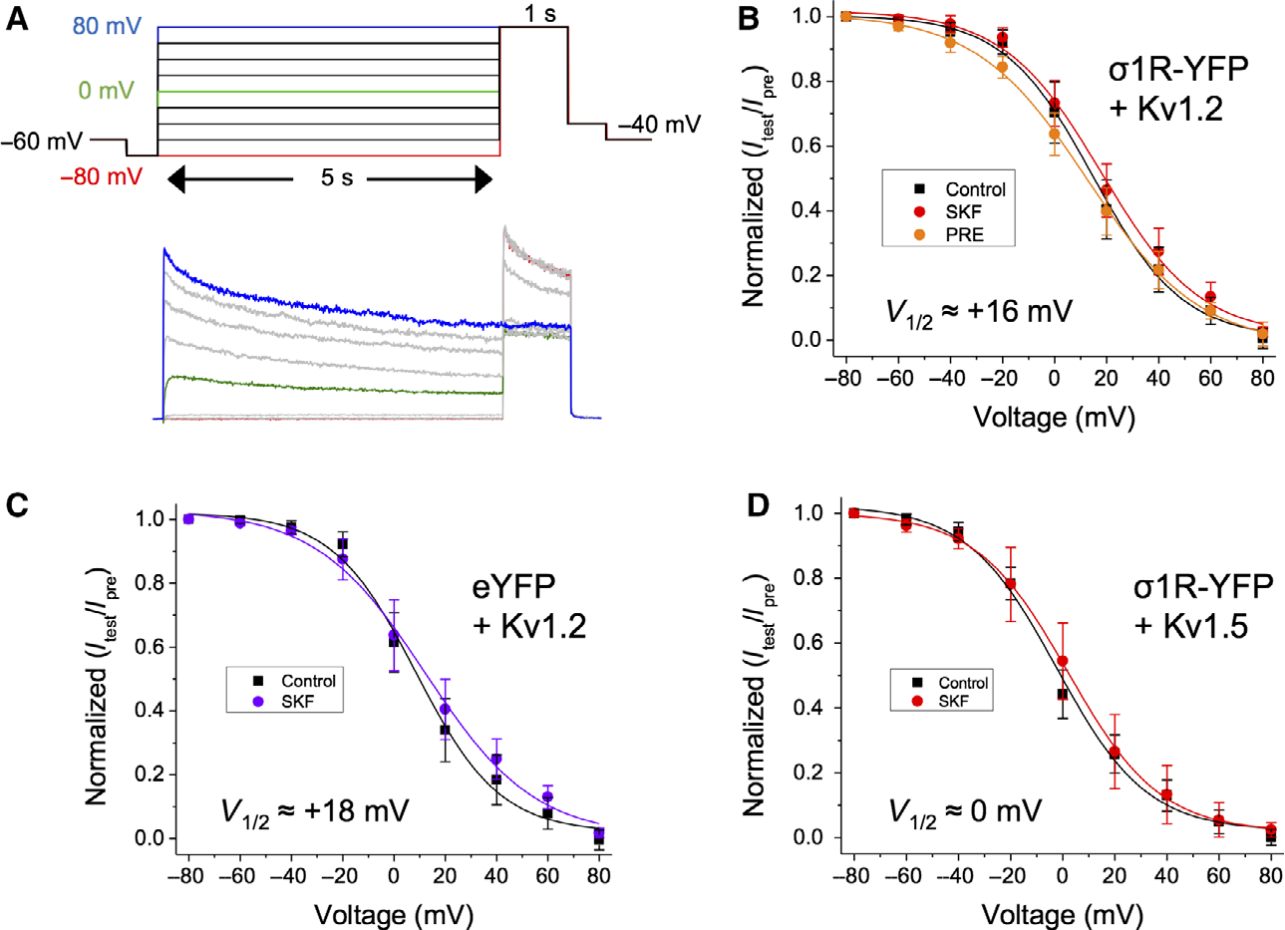
SKF has no effect on *V*_1/2_ of inactivation of Kv1.2. (A) Step protocol and a representative trace for determining the voltage dependency of inactivation for Kv1.2 channels. (B–D) Treatment with SKF caused no change in *V*_1/2_ of inactivation in cells expressing Kv1.2 and Sig‐1R‐YFP (B), or in cells expressing Kv1.2 and eYFP (C), or in cells expressing Kv1.5 and Sig‐1R‐YFP (D). Data are expressed as mean ± 95% CI. Curves shown are single Boltzmann fits to the averaged data unless otherwise stated. *V*_1/2_ values cited are derived from the mean of a Boltzmann fit to each individual cell in the dataset.

To test this hypothesis, we performed the electrophysiological protocol shown in Figure 4A on HEK293 cells transfected with Kv1.2 and either Sig‐1R‐YFP or eYFP, before and during bath administration of 50 *μ*mol/L SKF. Each cell was given a 5‐sec prepulse, ranging from −60 mV to +80 mV (in increments of 20 mV), followed by a 1 sec +80 mV test potential step (Fig. 4A). Voltage dependency of inactivation was determined by subtracting the 5 sec steady‐state current for each given prepulse potential from the peak current at the +80 mV test pulse. All values were normalized to +80 mV prepulse peak current. Normalized values were plotted against voltage and fitted with a single Boltzmann function to derive the *V*_1/2_ and slope of inactivation.

Cells co‐transfected with Kv1.2 and Sig‐1R‐YFP expressed Kv1.2 channels with a *V*_1/2_ of inactivation of 16.7 ± 4.76 mV and a slope (k) of 16.9 ± 2.22 mV in control conditions (Fig. 4B, black; *n* = 10). Bath application of SKF had no significant effect on either *V*_1/2_ (16.75 ± 3.97 mV) or on slope (16.97 ± 3.32 mV; Fig. 4B, red; *P* = 0.98; *n* = 10). Administration of PRE‐084 also had no significant effect on *V*_1/2_ (12.58 ± 2.17 mV) or on slope (18.44 ± 3.18 mV) of inactivation (Fig. 4B, orange; *P* = 0.86; *n* = 6). Similarly, in cells co‐transfected with Kv1.2 and eYFP, SKF had no significant effect on *V*_1/2_ or slope of inactivation (Fig. 4C, purple; *P* ~0.84; *n* = 8). Furthermore, cells transfected with Kv1.5 and Sig‐1R‐YFP showed no significant effect of SKF on *V*_1/2_ (~0 mV) or slope of inactivation (Fig. 4D; *P* = 0.71; *n* = 5). Thus, we show that Sig‐1R activation has no effect on the inactivation profile of Kv1.2 and Kv1.5, in contrast to other members of the *shaker* K+ channel family (Kinoshita et al. 2012).

### Kv1.2 exhibits predominantly “slow” activation gating when Sig‐1R is overexpressed

**Figure 5:**
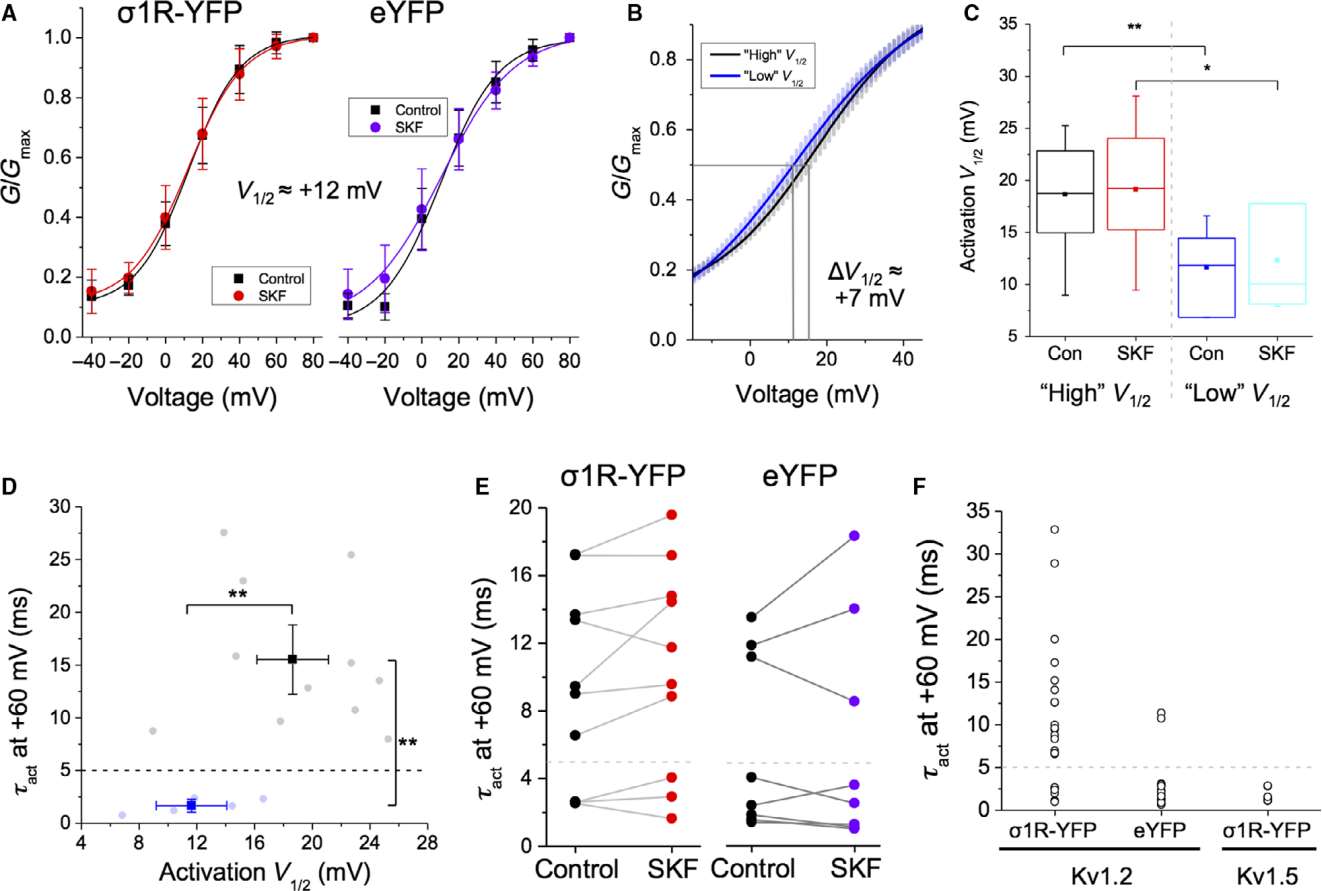
Co‐transfection of cells with Kv1.2 and Sig‐1R‐YFP leads to two populations of Kv1.2 channels, based on activation gating characteristics. (A) Bath application of SKF had no effect on the *V*_1/2_ of activation in cells expressing Kv1.2 and Sig‐1R‐YFP (A, red) or Kv1.2 and eYFP (A, purple). (B) However, in cells co‐transfected with Kv1.2 and Sig‐1R‐YFP, a “high” *V*_1/2_ (black) and a “low” *V*_1/2_ population of channels (blue) was observed with a *V*_1/2_ differential of ~7 mV (+18 mV c.f. +11 mV). Curve fits show mean ± SEM for each population of channels. (C) Box and whisker plot shows that while a significant decrease in *V*_1/2_ was observed between “high” and “low” *V*_1/2_ channels, SKF had no additional effect. (D–F) Plotting *V*_1/2_ of activation against *τ*_act_ more clearly reveals the two distinct channel populations and reveals a “cutoff” *τ*_act_ for “fast” and “slow” activating channels ~5 msec (D). These two populations were also observed in cells transfected with Kv1.2 and eYFP (E, purple dots), and it was found that SKF has no significant effect on *τ*_act_ of either population (E). A scatterplot of *τ*_act_ against cDNA transfected shows that 67% of cells transfected with Kv1.2 and Sig‐1R‐YFP (i.e., overexpressing Sig‐1R) have channels in the “slow” mode (F, left column). Only 17% of channels are in the “slow” mode (F, middle) in cells transfected with Kv1.2 and eYFP (i.e., endogenous Sig‐1R expression). Cells transfected with Kv1.5 and Sig‐1R‐YFP are 100% “fast” (F, right column). Data are expressed as mean ± 95% CI, except for scatterplots where each point represents a channel population sampled from a single cell. In box and whisker plots, boxes represent data between first and third quartile, while whiskers represent 1.5 × IQR. Curves shown are single Boltzmann fits to the averaged data unless otherwise stated. *V*_1/2_ values cited are derived from the mean of a Boltzmann fit to each individual cell in the dataset. Asterisks indicate statistical significance; single asterisks (*) represent *P* < 0.05, while double asterisks (**) indicate *P* < 0.005.

We next investigated whether Sig‐1R had a role in modulating the voltage dependence and kinetics of Kv1.2 channel activation. To do this, we converted the peak current at membrane potentials between −40 and +80 mV (+20 mV increments) into conductance (G), normalizing relative to the maximal conductance (G_max_) at +80 mV, before fitting the data with a single Boltzmann sigmoid (Fig. 5A). The *V*_1/2_ of activation of Kv1.2 co‐expressed with either Sig‐1R‐YFP (Fig. 5A, red) or with eYFP (Fig. 5A, purple) was 15.2 ± 1.9 mV (*n* = 19) and 10.5 ± 4.97 mV (*n* = 8), respectively. In both cases, there was no significant change in the *V*_1/2_ of activation in the presence of SKF (*P* ~0.98 for Sig‐1R‐YFP or eYFP). However, deeper examination of the data revealed two distinct populations of channels when Kv1.2 was co‐expressed with Sig‐1R‐YFP (Fig. 5B), a “high” *V*_1/2_ (18.64 ± 2.47 mV; *n* = 12) and “low” *V*_1/2_ (11.62 ± 2.45 mV; *n* = 7) population of channels. There was a significant difference between the *V*_1/2_ of activation between “high” *V*_1/2_ and “low” *V*_1/2_ channels (Fig. 5C; *P* = 0.006; *n* = 7–12). However, no significant effect of SKF on the voltage dependence of activation for either of these channel populations was observed (Fig. 5C; *P* ~0.83; *n* = 7–12).

In addition, the “high” *V*_1/2_ channels had different activation kinetics compared to the “low” *V*_1/2_ channels. Specifically, the “high” *V*_1/2_ channels had slower activation time courses that were best fit with a double exponential function, in contrast to “low” *V*_1/2_ channels, which had fast activation kinetics best described with a single exponential. To facilitate comparison between these two populations of channels, we plotted *V*_1/2_ of activation against activation tau (*τ*_act_) at +60 mV using a weighted exponential function for the “high” *V*_1/2_ channels (Fig. 5D). We observed a clear difference in *τ*_act_ between both populations – “high” *V*_1/2_ channels had a mean weighted *τ*_act_ of 15.5 ± 4.29 msec, while “low” *V*_1/2_ channels had a *τ*_act_ of 1.67 ± 0.61 msec (Fig. 5D; *P* = 0.0005; *n* = 7–12). Interestingly, no Kv1.2 channels with *τ*_act_ value between ~4–6 msec were observed (Fig. 5D). Thus, we were able to clearly define “slow” channels as those having a *τ*_act_ >5 msec, while “fast” channels had a *τ*_act_ of <5 msec (Fig. 5D–F, dashed gray line), as opposed to using the “high *V*_1/2_″ and “low *V*_1/2_” monikers.

In cells co‐transfected with Kv1.2 and Sig‐1R‐YFP, 16/24 (67%) of cells had channels that were “slow,” with the remainder (33%) being “fast.” There was no effect of SKF on activation kinetics in either “fast” or “slow” channel population (Fig. 5E, right; *P* = 0.71; *n* = 8). In contrast, there were markedly less “slow” channels in cells co‐transfected with Kv1.2 and eYFP (Fig. 5E–F). Here, only 3/18 cells (17%) had “slow” channels (*τ*_act_ = 11.68 ± 1.26 msec), with the remainder (83%) being “fast” (*τ*_act_ = 2.01 ± 0.52 msec; Fig. 5F). Again, SKF had no effect on the *τ*_act_ of either channel population (Fig. 5E, left; *P* = 0.11; *n* = 10). This effect appears to be specific to the Kv1.2 subtype, since 12/12 (100%) of cells transfected with Kv1.5 and Sig‐1R‐YFP expressed “fast” channels, with a *τ*_act_ of 1.51 ± 0.36 msec (Fig. 5F).

It is known that Kv1.2 has two distinct activation‐gating modes, “fast” and “slow” (Rezazadeh et al. 2007; Baronas et al. 2015). We show that gating kinetics of these modes is coupled to the *V*_1/2_ of activation; “slow” channels have a more depolarized *V*_1/2_ of activation than “fast” channels. We further show that the propensity of Kv1.2 to exist in one of these two gating modes is dependent on the level of Sig‐1R expression. Overexpression of Sig‐1R increases the proportion of Kv1.2 channels that exist in the “slow” gating mode. In contrast, cells expressing eYFP (i.e., endogenous Sig‐1R expression levels) appear to have a higher proportion of Kv1.2 channels in the “fast” gating mode. These results are a new indication that the level of expression of Sig‐1R may alter the functionality of Kv1.2 channels.

### “Slow” Kv1.2 channels exhibit bimodal activation gating

Unique within the Kv1.x channel family, Kv1.2 is known to display bimodal activation gating (Grissmer et al. 1994; Rezazadeh et al. 2007). Although the associated signaling molecules have not yet been identified, it is known that an intracellular threonine residue (Thr252) in the S2–S3 linker of Kv1.2 is responsible for “slow” gating behavior (Rezazadeh et al. 2007). It has been suggested that a cytoplasmic extrinsic regulator (Rezazadeh et al. 2007) may bind to Kv1.2 in the closed state (Baronas et al. 2016) to regulate activation gating and use‐dependent activation. As co‐expression of Kv1.2 and Sig‐1R appears to produce two distinct Kv1.2 channel populations each with distinct activation parameters, we hypothesized that these two channel populations are due to Kv1.2 channels existing in either the “slow” gating mode or the “fast” gating mode. Furthermore, as Sig‐1R overexpression results in an increase in the proportion of “slow” channels in the population (Fig. 5F), we also hypothesized that changes in Sig‐1R expression levels relative to Kv1.2 could alter the gating mode of the channel.

**Figure 6:**
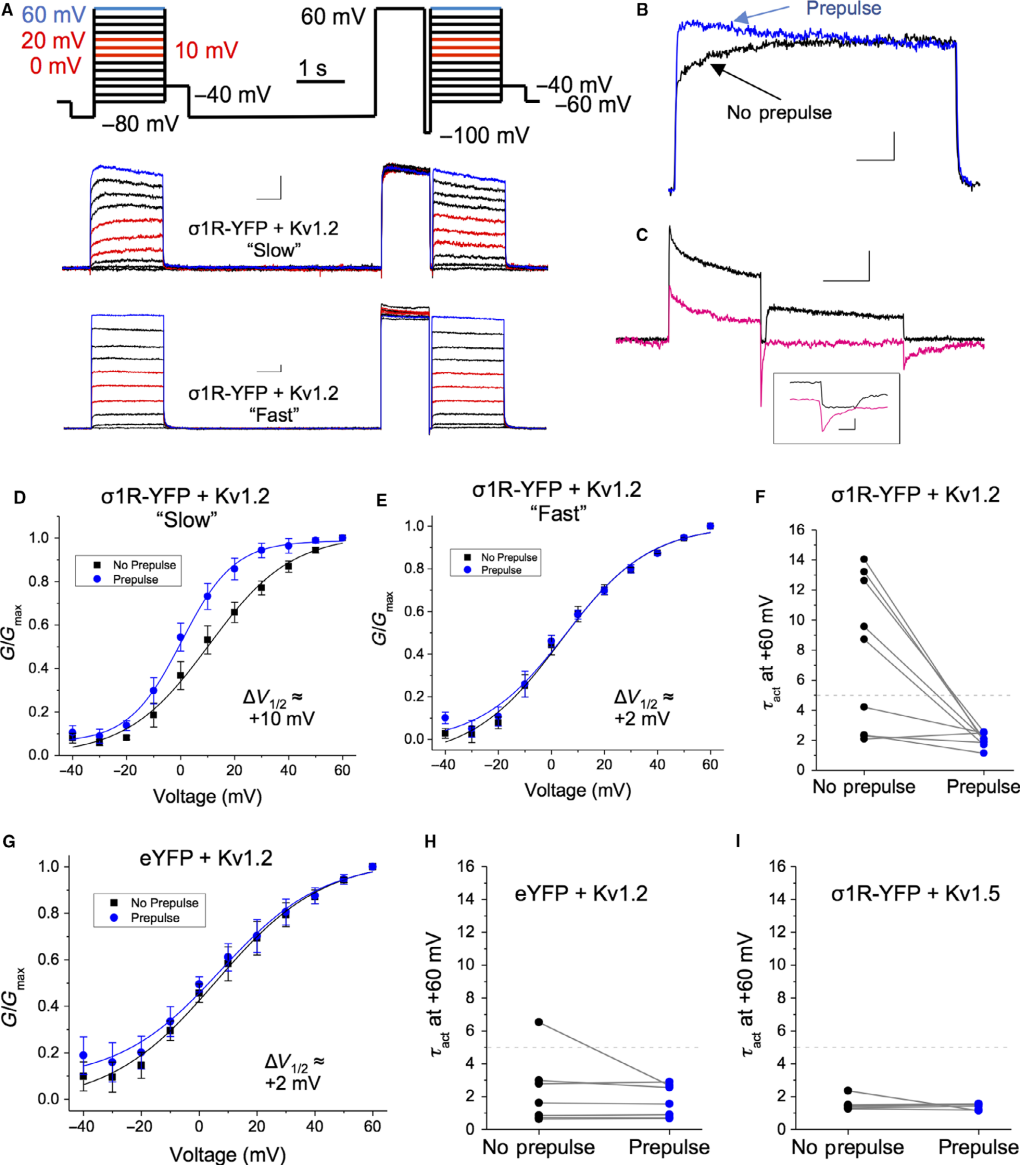
A “mode switching” protocol reliably recapitulates the two distinct populations of Kv1.2 channels observed following overexpression of Sig‐1R. (A) Step protocol for determining Kv1.2 activation gating mode (top) and for switching between “slow” and “fast” gating modes, with representative sample traces for “slow” (middle) and “fast” cells (bottom). (B) Superimposed traces illustrating that the “mode switch” induced by the prepulse can be clearly identified by the acceleration of *τ*_act_. (C) Representative traces of the hyperpolarizing step to −100 mV in normal extracellular K^+^ (3 mmol/L KCl; black trace) or in symmetrical KCl (135 mmol/L; pink trace) show that this “mode switch” is not due to incomplete deactivation following the prepulse. In symmetrical KCl, the *τ*_deact_ is ~12 msec, while the duration of the step is > 3 × *τ*_deact_. (D–F) Channels in the “slow” gating mode showed a leftward shift in voltage dependency of activation following the prepulse (D), while channels in the “fast” gating mode showed no significant shift (E). The prepulse resulted in a dramatic increase in the *τ*_act_ of “slow” channels, while the *τ*_act_ of the “fast” channels was largely unaffected (F). (G–H) In contrast, the prepulse had little effect on Kv1.2 channel gating in cells transfected with Kv1.2 and eYFP (G), as most of them were in the “fast” gating mode (H). (I) There was also no prepulse effect on the *τ*_act_ of Kv1.5 channels co‐transfected with Sig‐1R‐YFP. Data are expressed as mean ± 95% CI, except for scatterplots where each point represents a channel population sampled from a single cell. Curves shown are single Boltzmann fits to the averaged data unless otherwise stated. *V*_1/2_ values cited are derived from the mean of a Boltzmann fit to each individual cell in the dataset.

To test these hypotheses, we adapted a protocol used by Rezazadeh et al. (2007). Here, cells were stepped from −60 to +60 mV (Δ10 mV, 1.5 sec step duration) before being held at −80 mV for 4 sec. This was followed by a 1 sec step to +60 mV and a 50 msec step to −100 mV, before a second series of voltage steps between −60 and +60 mV were given (Fig. 6A). The first series of steps was termed “no prepulse,” while the second series of steps was termed “prepulse” following nomenclature proposed in Rezazadeh et al. (2007). Peak amplitudes from both the “no prepulse” and the “prepulse” steps were converted to conductance (G) to generate activation curves. The data obtained were fitted with a single Boltzmann function to derive *V*_1/2_ of activation for both groups independently.

As expected, we observed two distinct populations of channels from cells co‐transfected with Kv1.2 and Sig‐1R‐YFP – those with “slow” activation gating and those with “fast” activation gating. We were also able to convert “slow” channels into “fast” channels using the prepulse, as evidenced by the change in activation gating kinetics before the prepulse (Fig. 6B, black) and after the prepulse (Fig. 6B, blue). This was not due to incomplete deactivation of the channel in the interval between the prepulse and the second IV plot, as the *τ*_deact_ at ‐100 mV was 12.7 ± 3.23 msec when measured in symmetrical K^+^ solutions (135 mmol/L KCl; Fig. 6C, pink line). In cells that have Kv1.2 channels that exhibit “slow” gating, the *V*_1/2_ of activation of the “no prepulse” curve was 10.5 ± 2.94 mV (Fig. 6D). Following the prepulse, a ~10 mV leftward shift was observed (+0.28 ± 1.85 mV; *P* = 0.004; *n* = 5). In contrast, “fast” channels did not exhibit this phenomenon (Fig. 6E). Furthermore, “fast” channels did not show any acceleration in decay kinetics when measured before or after the prepulse (Fig. 6E), while the *τ*_act_ of the “slow” channels decreased from 11.63 ± 1.83 msec to 2.07 ± 0.23 msec after the prepulse (Fig. 6F; *P* < 0.01; *n* = 5).

In cells transfected with Kv1.2 and eYFP, we observed predominantly “fast” Kv1.2 channels, which did not exhibit a significant leftward shift in *V*_1/2_ of activation following the prepulse (Fig. 6G). Indeed, only one of the eight cells (12.5%) had Kv1.2 channels with a *τ*_act_ greater than 5 msec before the prepulse, which accelerated after the prepulse, as expected (Fig. 6H). Repeating these experiments on cells co‐transfected with Kv1.5 and Sig‐1R‐YFP, resulted in channels with activation kinetics ~2 msec, which were unaffected by the prepulse (Fig. 6I).

Taken together, we show that when the level of Sig‐1R expression is elevated, there is a tendency for Kv1.2 to exhibit slower activation kinetics and a more depolarized *V*_1/2_ of activation than when the level of Sig‐1R expression is low. This is not due to the genesis of two distinct populations of Kv1.2 channels as “slow” channels can be converted to “fast” channels by a prepulse. Rather, these experiments demonstrate that the expression level of the Sig‐1R is able to influence the gating pathway taken by the Kv1.2 channel in response to membrane depolarization and thereby regulate the gating kinetics of the channel.

### Expression of Kv*β*2 blocks the effect of SKF on Kv1.2, but has no effect on activation gating

The Kv*α* pore‐forming subunit of Kv1.x channels can interact with regulatory Kv*β* subunits that modulate channel inactivation and cell surface expression (Shi et al., 1996), via an interaction with the T_1_ domain of the Kv*α* subunit (Rettig et al. 1994; Accili et al. 1997b). We next sought to determine whether we could occlude the effects of either Sig‐1R pharmacological activation on Kv1.2 current amplitude or of Sig‐1R overexpression on Kv1.2 activation, via the expression of a Kv*β* subunit. Three subtypes of Kv*β* subunits have been identified (Rettig et al. 1994; Heinemann et al. 1996; Nakahira et al. 1996; Accili et al. 1997b), and while the Kv*β*1 is the best characterized of the Kv*β* subunits, they typically confer rapid inactivation in Kv1.x channels (Heinemann et al. 1996; Accili et al. 1997a), which may mask the effects of Sig‐1R activation. Thus, we chose to use Kv*β*2, which directly associates with Kv1.2 in the brain (Rhodes et al. 1997). More importantly, co‐expression of Kv*β*2 with Kv1.2 has little change in channel kinetics from that observed when Kv1.2 is expressed alone (Lazaroff et al. 2002).

**Figure 7:**
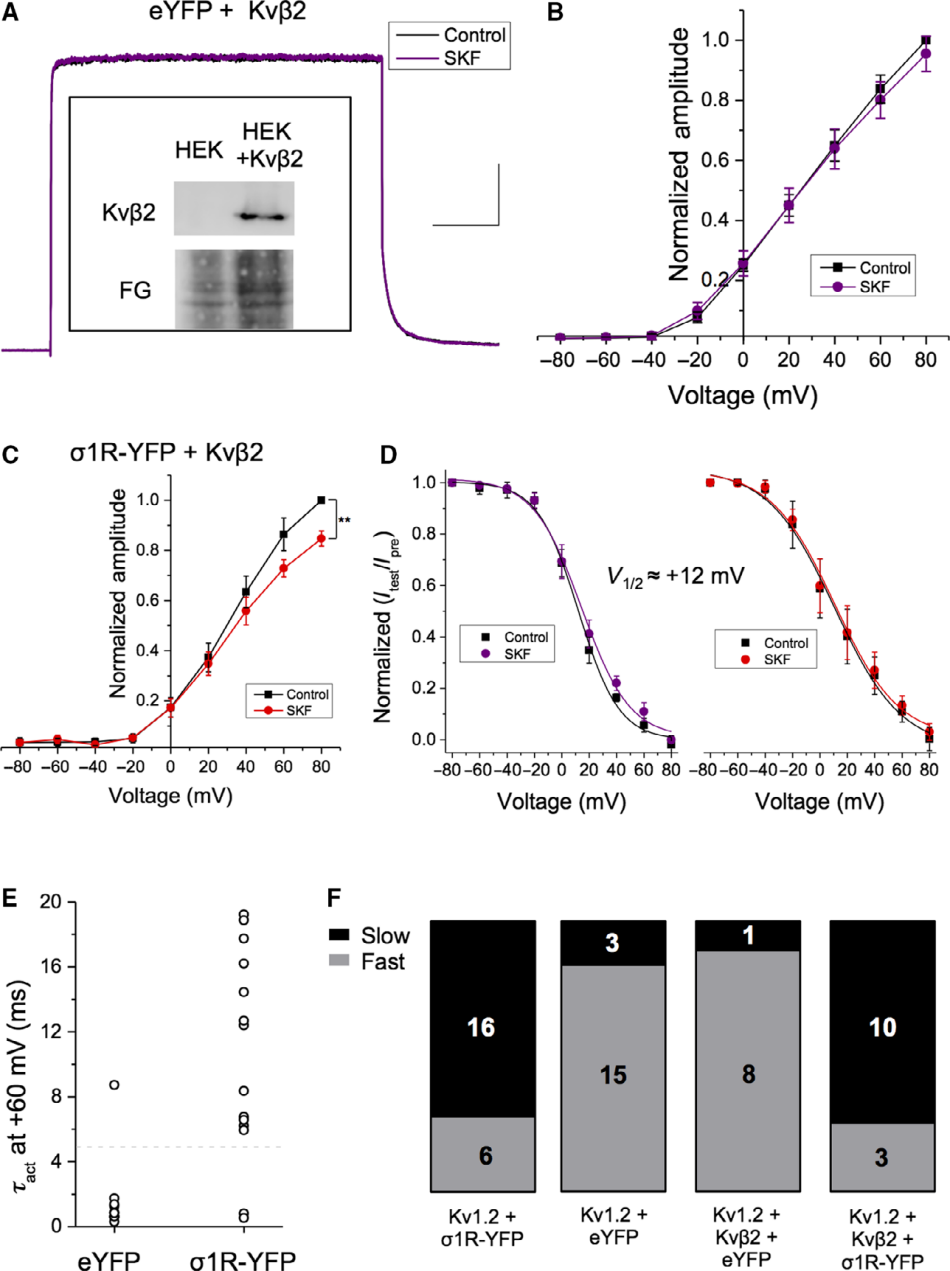
Expression of Kv*β*2 blocks the effect of Sig‐1R ligand activation on Kv1.2 current amplitude, except when Sig‐1R is overexpressed. (A–B) Western blotting displays expression of Kv*β*2 only in HEK293 cells transiently transfected with the cDNA encoding for that subunit (inset). Representative voltage‐clamp recordings of cells transfected with Kv1.2, Kv*β*2, and eYFP displayed no significant change (A) in current amplitude upon treatment with SKF at any membrane voltage tested (B, purple). Scale bar in Panel A is 200 msec and 300 pA. (C) In contrast, cells transfected with Kv1.2, Kv*β*2, and Sig‐1R‐YFP displayed a significant decrease in current amplitude upon treatment with SKF. (D) There was no significant effect of SKF in *V*_1/2_ of inactivation. (E–F) Only 11% of cells transfected with Kv1.2, Kv*β*2, and eYFP expressed Kv1.2 channels in the “slow” gating mode. In contrast, cells that overexpressed both Sig‐1R‐YFP and Kv*β*2 had 77% of Kv1.2 channels in the “slow” gating mode. Data are expressed as mean ± 95% CI, except for scatterplots where each point represents a channel population sampled from a single cell. Curves shown are single Boltzmann fits to the averaged data unless otherwise stated. *V*_1/2_ values cited are derived from the mean of a Boltzmann fit to each individual cell in the dataset. Asterisks indicate statistical significance; single asterisks (*) represent *P* < 0.05, while double asterisks (**) indicate *P* < 0.005.

We first confirmed via Western blotting that HEK293 cells do not endogenously express Kv*β*2, and that transfection with Kv*β*2 cDNA would induce robust Kv*β*2 protein expression (Fig. 7A, inset). In electrophysiology experiments, application of SKF had no significant effect on current amplitude in cells transfected with Kv1.2, Kv*β*2, and eYFP (Fig. 7A and B, purple trace; *P* = 0.78; *n* = 8). These findings support the notion that the effect of SKF on Kv1.2 channels is mediated by the Sig‐1R and is not due to a direct interaction between SKF and Kv1.2.

However, cells transfected with Kv1.2, Sig‐1R‐YFP, and Kv*β*2 together exhibited an irreversible 15.3 ± 3.02% decrease in current amplitude at +80 mV upon application of SKF (Fig. 7C; *P* = 0.008; *n* = 6), which is intermediate to cells expressing Kv1.2 and Sig‐1R alone (~28% decrease in current amplitude) and cells expressing Kv1.2, eYFP, and Kv*β*2 (~4% decrease). Bath application of SKF had no effect on *V*_1/2_ and slope of inactivation in either cell population (Fig. 7C; *P* ~0.6; *n* = 6–8).

Cells co‐transfected with Kv1.2, eYFP, and Kv*β*2 had Kv1.2 channel populations exhibiting heterogenous activation gating (Fig. 7E and F). However, the vast majority of cells (8/9; 88%) exhibited “fast” activation gating, with a *τ*_act_ of 1.2 ± 0.22 msec at +60 mV. Only one sampled cell displayed “slow” gating, with an activation tau of 10.7 msec at +60 mV (Fig. 7E and F). As the effect of Sig‐1R activation on Kv1.2 current amplitude was partially restored in cells co‐transfected with Kv1.2, Kv*β*2, and Sig‐1R‐YFP, we hypothesized that “slow” activation gating would again be present in a proportion of the sampled cells. As predicted, we observed Kv1.2 channels exhibiting both “slow” and “fast” activation gating (Fig. 7E and F), with the “slow” channels having a *τ*_act_ of 11.24 ± 2.93 msec (*n* = 10) and the “fast” channels having a *τ*_act_ of 1.31 ± 0.76 msec (*n* = 3). The majority of channels (10/13, 77%) in cells co‐transfected with Kv1.2, Kv*β*2, and Sig‐1R‐YFP occupied the “slow” gating mode (Fig. 7F), a similar proportion to cells overexpressing Sig‐1R in the absence of Kv*β*2 (67%; Fig. 7F).

Our data show that co‐expression of Kv*β*2 with Kv1.2 attenuates the effects of Sig‐1R ligand activation on Kv1.2 channel conductance but does not affect activation gating. When Sig‐1R was co‐transfected with Kv*β*2 and Kv1.2, we found that the response of Kv1.2 to SKF was intermediate to that observed when Sig‐1R was singly transfected along with Kv1.2. This suggests that the presence of Kv*β*2 acts to inhibit the effect of a ligand‐activated Sig‐1R, possibly due to a competitive interaction between the two proteins in some form of macromolecular complex.

### Sig‐1R‐E102Q has decreased interaction with Kv1.2 compared to WT Sig‐1R

Mutations in the Sig‐1R are clinically associated with motor neuron pathologies such as distal hereditary motor neuropathy and amyotrophic lateral sclerosis (Luty et al. 2010; Al‐Saif et al. 2011; Li et al. 2015; Gregianin et al. 2016). Interestingly, Kv1.2 dysfunction or deletion is also associated with motor neuron disease, where it results in neuronal hyperexcitability (Brew et al. 2007; Shibuya et al. 2011; Robbins and Tempel 2012; Helbig et al. 2016). Therefore, in our final line of experimentation, we examined how modulation of Kv1.2 may be altered in cells expressing the mutant Sig‐1R underlying ALS16 (Sig‐1R‐E102Q; Al‐Saif et al. 2011). The Sig‐1R‐E102Q mutation results in the substitution of glutamic acid (E) for glutamine (Q) in the *β*‐barrel domain of the Sig‐1R (Schmidt et al. 2016). Although this mutation manifests as the loss of a single hydrogen bond in the final protein product, Sig‐1R‐E102Q differs dramatically from Sig‐1R‐WT in terms of subcellular localization and K^+^ channel modulation (Tagashira et al. 2014; Fukunaga et al. 2015; Shinoda et al. 2015; Wong et al. 2016; Dreser et al. 2017).

**Figure 8:**
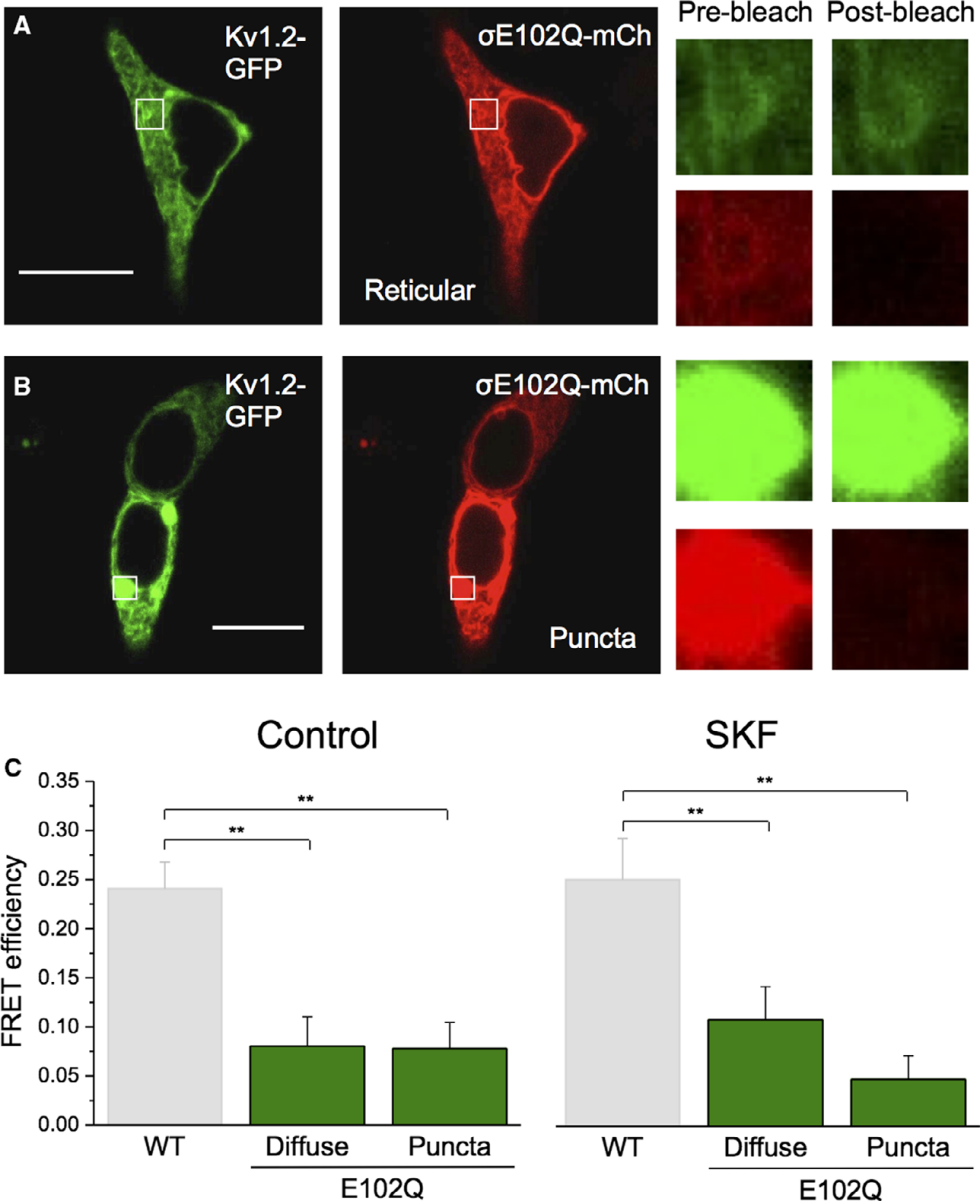
Sig‐1R‐E102Q shows decreased interaction with Kv1.2 as compared to WT Sig‐1R. (A–B) Representative confocal images of HEK293 cells transiently co‐expressing Kv1.2‐GFP and Sig‐1R‐E102Q‐mCh (σE102Q‐mCh), with reticular Sig‐1R localization (A) and Sig‐1R aggregation into puncta (B), before and after acceptor photobleaching. Scale bars in Panels A and B represent 10 *μ*m. (C) Quantification of the images shows a significant decrease of FRET efficiency in both Sig‐1R‐E102Q‐mCh groups as compared to WT Sig‐1R‐mCh and no significant differences between reticular and puncta Sig‐1R‐E102Q cells. There was no significant effect of SKF on FRET efficiency of either reticular or puncta Sig‐1R‐E102Q‐mCh. Data are expressed as mean ± 95% CI. Double asterisks (**) represent *P* < 0.005.

Confocal imaging experiments on HEK293 cells transfected with Sig‐1R‐E102Q‐mCh show a strikingly different localization pattern compared with cells transfected with Sig‐1R‐WT‐mCh. While the WT Sig‐1R shows reticular patterning that is localized to the ER, Sig‐1R‐E102Q has decreased reticular patterning, aggregates in large puncta, and exhibits a small amount of diffuse distribution within the nucleus (Fig. 8A and B). We performed apFRET experiments on both reticular Sig‐1R‐E102Q (Fig. 8A) and Sig‐1R‐E102Q puncta (Fig. 8B) using Kv1.2 as the fluorescence donor and speculated that dissimilar subcellular localization of Sig‐1R‐E102Q may indicate variability in the interaction with Kv1.2 channels.

In cells with reticular Sig‐1R‐E102Q‐mCh expression, it was found that FRET efficiency was 8.1 ± 1.8% (Fig. 8A and C; *n* = 29), which was a significant decrease compared to the FRET efficiency of cells expressing Sig‐1R‐WT (~22%; *P* = 3.6 × 10^−10^). Although Sig‐1R‐E102Q‐mCh puncta consistently colocalized with Kv1.2‐GFP puncta, FRET efficiency was only 6.04 ± 0.81% (Fig. 8B and C; *P* = 1.4 × 10^−11^ relative to Sig‐1R‐WT; *n* = 20). However, there was no significant difference in FRET efficiency between Kv1.2‐GFP and either reticular or puncta Sig‐1R‐E102Q‐mCh (*P* = 0.31), indicating that both groups similarly interact with Kv1.2‐GFP. There was also no significant difference in FRET efficiency in either group when SKF was bath applied for 20 min (Fig. 8D; *P* ~0.2 for both groups). These data demonstrate that Sig‐1R‐E102Q‐mCh shows decreased interaction with Kv1.2‐GFP compared to Sig‐1R‐WT‐mCh in baseline conditions and upon agonist application.

### Expression of Sig‐1R‐E102Q modulates inactivation and activation gating of Kv1.2

**Figure 9:**
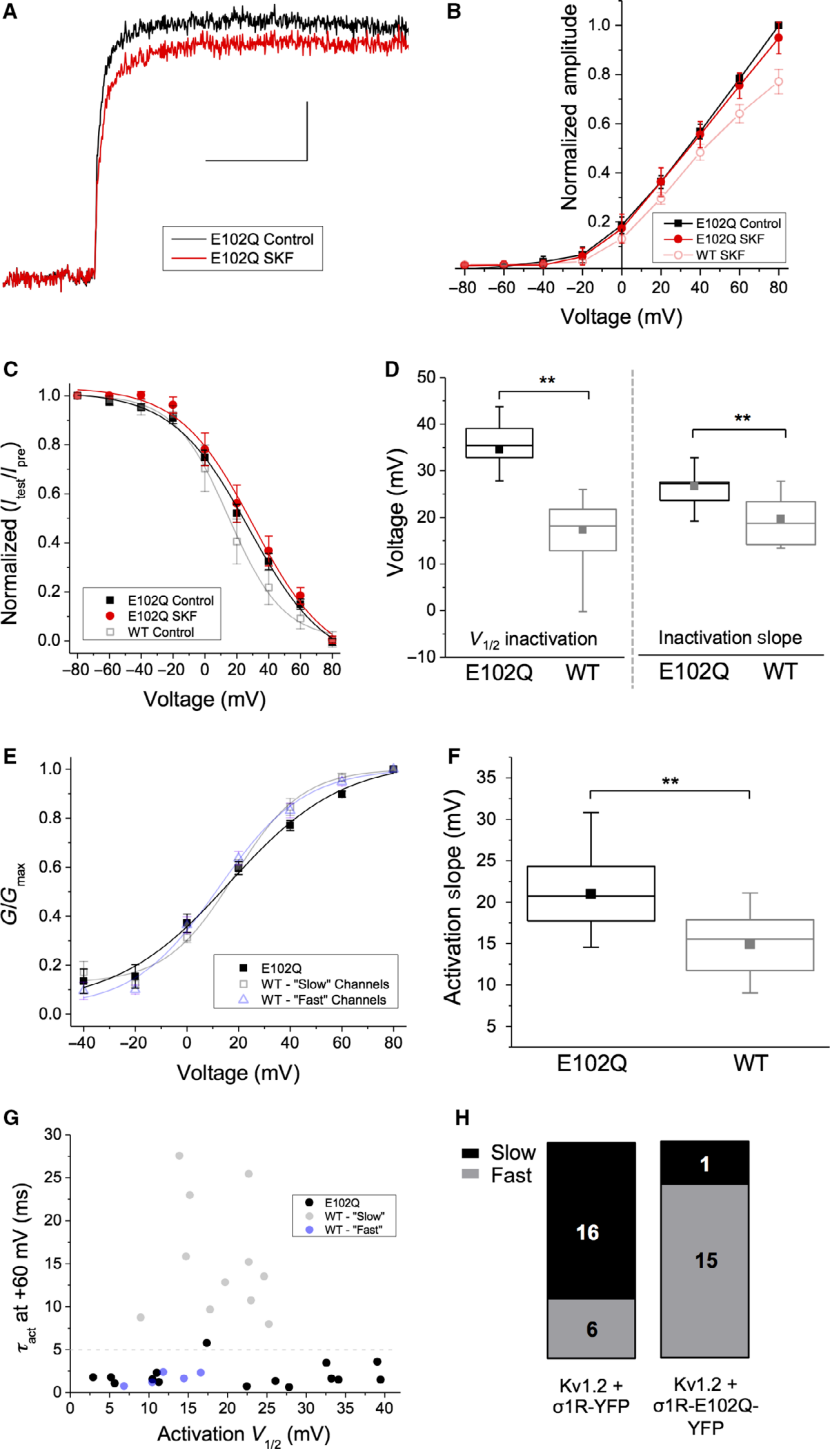
Expression of Sig‐1R‐E102Q abolishes the effect of SKF on Kv1.2 current amplitude and decouples the relationship between slow *τ*_act_ and “high” *V*_1/2_ of Kv1.2 channels in the “slow” gating mode. (A) Representative trace from a cell co‐transfected with Kv1.2 and Sig‐1R‐E102Q‐YFP (σE102Q‐YFP) in response to a depolarizing step from −80 to +80 mV in control conditions (black) and in the presence of SKF (red). Scale bar is 100 msec and 300 pA. (B–C) There was no significant decrease in current amplitude upon treatment with SKF at any voltage tested (B, red) or on *V*_1/2_ of inactivation (C, red). (D–F) Box and whisker plots demonstrating that cells transfected with Sig‐1R‐E102Q‐YFP had Kv1.2 channels with a significantly right‐shifted *V*_1/2_ (D, left) and slope of inactivation (D, right) compared to those transfected with Sig‐1R‐WT‐YFP. Sig‐1R‐E102Q‐YFP cells also expressed Kv1.2 channels with a significantly shallower activation slope than observed in cells transfected with Sig‐1R‐WT‐YFP (F). (G–H) The relationship between *V*_1/2_ of activation and *τ*_act_ observed in Kv1.2 channels in cells overexpressing with Sig‐1R‐WT‐YFP (G, transparent black and blue dots), is abolished in cells overexpressing Sig‐1R‐E102Q‐YFP (G, black dots). Although a widespread of *V*_1/2_ of channel activation is observed in these cells, the *τ*_act_ remains ≤ 5 msec irrespective of *V*_1/2_, with only one cell being classed as “slow” with a *τ*_act_ of ~5.5 msec (H). Data are expressed as mean ± 95% CI, except for scatterplots where each point represents a channel population sampled from a single cell. Curves shown are single Boltzmann fits to the averaged data unless otherwise stated. *V*_1/2_ values cited are derived from the mean of a Boltzmann fit to each individual cell in the dataset. In box and whisker plots, boxes represent data between first and third quartile, while whiskers represent 1.5 × IQR. Asterisks indicate statistical significance; single asterisks (*) represent *P* < 0.05, while double asterisks (**) indicate *P* < 0.005.

As Sig‐1R‐E102Q has decreased interaction with Kv1.2 compared to Sig‐1R‐WT, we speculated that this would affect the functional modulation of Kv1.2 channels. Therefore, we characterized Kv1.2 channels in cells co‐transfected with equimolar amounts of Kv1.2 and Sig‐1R‐E102Q‐YFP. Application of SKF had no significant effect on current amplitude in these cells (5.1 ± 7.3% decrease at +80 mV; Fig. 9A and B; *P* = 0.87; *n* = 5), in contrast to cells transfected with Sig‐1R‐WT and Kv1.2, where a ~28% decrease in current amplitude is observed (Fig. 9B, open red transparent circles).

We observed a significant rightward shift in the *V*_1/2_ of inactivation in cells co‐transfected with Kv1.2 and Sig‐1R‐E102Q‐YFP (28.4 ± 4.69 mV) compared with those co‐transfected with Kv1.2 and Sig‐1R‐YFP (17.3 ± 4.79 mV; Fig. 9C, open black transparent squares; *P* < 0.01; *n* > 16). This increase in *V*_1/2_ of inactivation was also accompanied by a significant increase in inactivation slope from 16.6 ± 1.75 mV when Sig‐1R‐YFP was expressed, to 28.4 ± 4.68 mV in the presence of Sig‐1R‐E102Q‐YFP (Fig. 9D; *P* < 0.01; *n* > 16). There was no significant effect of SKF on the *V*_1/2_ of inactivation in cells expressing Sig‐1R‐E102Q‐YFP (Fig. 9C, red; *P* = 0.94; *n* = 5).

Moreover, we observed no change in overall *V*_1/2_ of activation when Sig‐1R‐E102Q‐YFP was co‐expressed with Kv1.2 compared with Sig‐1R‐YFP (Fig. 9E, black). However, expression of Sig‐1R‐E102Q‐YFP resulted in an activation slope of 21.0 ± 2.17 mV (*n* = 16), a significant rightward shift from an activation slope of 14.5 ± 2.10 mV (*n* = 24) observed when Sig‐1R‐YFP was co‐transfected with Kv1.2 (Fig. 9E and F; *P* < 0.01). Furthermore, we were also not able to clearly discern “slow” and “fast” Kv1.2 channels based on *τ*_act_ or *V*_1/2_ in the overall population of cells co‐transfected with Kv1.2 and Sig‐1R‐E102Q‐YFP (Fig. 9G). While a clear separation in *V*_1/2_ of activation was evident (Fig. 9G, black dots), the vast majority (15/16; 93%) of the channels had “fast” activation gating with a *τ*_act_ 1.53 ± 0.36 msec at +60 mV. Only one cell expressed channels that could be classified as “slow,” having a *τ*_act_ of 5.25 msec at +60 mV (Fig. 9H).

These data demonstrate that expression of Sig‐1R‐E102Q affects inactivation and activation gating of Kv1.2, which is not observed when cells express Sig‐1R‐WT. The effect of SKF on Kv1.2 current amplitude is also abolished in Sig‐1R‐E102Q expressing cells. In addition, while overexpression of WT Sig‐1R increases the proportion of channels displaying “slow” activation gating, overexpression of Sig‐1R‐E102Q results in the vast majority of channels exhibiting “fast” activation gating and decouples the relationship between *V*_1/2_ and *τ*_act_. Overall, these results are an indication that Sig‐1R‐E102Q can modulate multiple functional properties of Kv1.2 and that pharmacological activation of Sig‐1R‐E102Q has no additional effects.

## Discussion

This study represents the first biophysical characterization of Kv1.2 modulation by Sig‐1R. We found that application of Sig‐1R agonist decreases Kv1.2 current amplitude, likely due to a ligand‐dependent change in Sig‐1R activity or conformation rather than increased association of Sig‐1R with Kv1.2, as the effect of Sig‐1R agonist application is abolished in the presence of Kv*β*2. We also show that overexpression of Sig‐1R results in Kv1.2 channels that preferentially exhibit “slow” activation gating, characterized by slower kinetics and a more depolarized *V*_1/2_ of activation. Kv1.2 channels that exist in the “slow” gating mode can be shifted into the “fast” gating mode by a prepulse, demonstrating that Sig‐1R is able to modulate Kv1.2 activation gating. Expression of Sig‐1R‐E102Q abolishes Sig‐1R agonist modulation of Kv1.2 and leads to a rightward shift in the voltage dependence of inactivation and activation. Furthermore, the presence of Sig‐1R‐E102Q decouples the relationship between activation kinetics and *V*_1/2_ of activation, resulting in Kv1.2 channels exclusively existing in the “fast” gating mode.

### Ligand‐dependent regulation of Kv1.2 by Sig‐1R

There is a large body of evidence demonstrating that ligand activation of Sig‐1R consistently inhibits potassium channels in native and recombinant systems (Soriani et al. 1999; Wilke et al. 1999; Lupardus et al. 2000; Aydar et al. 2002; Zhang and Cuevas 2005; Martina et al. 2007; Kinoshita et al. 2012; Wong et al. 2016). In this work, we show that Sig‐1R activation by SKF inhibits Kv1.2, as observed for other Kv1.x subtypes (Aydar et al. 2002; Kinoshita et al. 2012; Kourrich et al. 2013). This is likely due to Sig‐1R activation rather than a direct effect of the ligand on the channel (Lamy et al. 2010; Liu et al. 2017) as this effect is abolished in the presence of Kv*β*2 and Sig‐1R‐E102Q.

Although this study was performed in a recombinant system, the interaction between the Sig‐1R and Kv1.2 is a conserved cellular mechanism which extends to native systems (Delint‐Ramirez et al. 2018); thus, we can speculate that our identified regulatory mechanisms will extend to neuronal cells. Of note, treatment with cocaine (a noncanonical Sig‐1R agonist) promotes the interaction between Sig‐1R and Kv1.2 in nucleus accumbens neurons (Kourrich et al. 2013), but we show that treatment with SKF (a canonical Sig‐1R agonist) does not change the interaction between Sig‐1R and Kv1.2. The disparity could be due to the duration of ligand application, as Kourrich et al. performed their study in the context of prolonged treatment, while our study examined acute ligand application. Alternately, there may be distinct changes in Sig‐1R conformation following binding of different Sig‐1R ligands, which may lead to different downstream effects. Binding of cocaine only requires the presence of D188 and the last 16 C‐terminal amino acid residues of the Sig‐1R (Chen et al. 2007; Brune et al. 2013; Delint‐Ramirez et al. 2018). The more bulky Sig‐1R ligands, such as SKF, contact amino acids further upstream from D188 (Brune et al. 2014), which may aid in the stabilization of these molecules in the large binding pocket (Schmidt et al. 2016). Thus, Sig‐1R could adopt distinct conformations depending on the ligand which is bound (Gromek et al. 2014; Mishra et al. 2015).

The fact that Sig‐1R can directly interact with Kv1.2 and modify channel function is reminiscent of Kv*β* modulation of Kv1.x channels (Rettig et al. 1994; Pongs et al. 1999; Pongs and Schwarz 2010). Although the Sig‐1R and Kv*β* subunits are faithful modulators of Kv1.x channels, Sig‐1R remains distinct and unique in its mode of action. First, Sig‐1R modulates a multitude of ion channels in addition to Kv1.x, while Kv*β* subunits are specific to Kv1.x channels (Heinemann et al. 1996; Sewing et al. 1996) due to the high specificity of the Kv*α*‐Kv*β* contact loop (Gulbis et al. 2000). Second, Sig‐1R regulation of Kv1.x channels appears to be dynamic and subtype specific, while Kv*β* subunits exert predictable effects on Kv1.x channel conductance and inactivation (Heinemann et al. 1996). Finally, Sig‐1R interacts with Kv1.3 at a locus within the transmembrane domain region (Kinoshita et al. 2012), while Kv*β* subunits exert their effects by binding to the Kv1.x N‐terminal T_1_ domain upstream of the transmembrane domain (Rettig et al. 1994). Deletion of the N‐terminal domain of Kv1.3, including the Kv*β* binding site, has no effect on Sig‐1R binding (Kinoshita et al. 2012), arguing against a common binding site within Kv1.x channels for Kv*β* and Sig‐1R. Our data showing that the presence of Kv*β*2 blocks agonist‐induced Sig‐1R modulation of Kv1.2 suggests that the Kv*β* subunit prevents Sig‐1R from adopting a ligand‐activated conformation. This could occur by physical occlusion or perhaps following structural rearrangements of the Kv*α* subunit following Kv*β* binding (Sokolova et al. 2003).

### Ligand‐independent regulation of Kv1.2 by Sig‐1R

Previous work has shown that the Sig‐1R resides in the ER membrane where it is clustered at ER specializations juxtaposed to mitochondria (Hayashi and Su 2007), and the plasma membrane (Mavlyutov et al. 2015, 2016, 2017; Wong et al. 2016). Additionally, it has been shown that the Sig‐1R can translocate to the nuclear envelope upon activation with cocaine, and it has also been reported that the Sig‐1R may reside in the PM in dorsal root ganglion (DRG) neurons (Mavlyutov et al. 2015; Tsai et al. 2015). The crystal structure of the Sig‐1R suggests that the large C‐terminal domain of the receptor is on the cytosolic face of the ER membrane (Schmidt et al. 2016). However, more recently, APEX2‐electron microscopy experiments in primary neurons and in vivo DRG neurons convincingly determine that the large C‐terminal domain of the Sig‐1R resides in the ER lumen (Mavylutov et al. 2017). While our experiments did not address these questions directly, our data may lend support to the hypothesis that the Sig‐1R may translocate to the PM in order to interact with PM‐resident ion channels.

The functional characteristics of PM ion channels are largely unaffected by Sig‐1R expression level (reviewed by Kourrich et al. 2012). However, co‐expression of Kv1.3 and Sig‐1R accelerates channel inactivation in the absence of Sig‐1R ligands (Kinoshita et al. 2012). This is also observed for Kv1.4; however, the acceleration in channel inactivation with increasing Sig‐1R expression levels was also accompanied by a progressive decrease in K^+^ current amplitude (Aydar et al. 2002). Our data show that Sig‐1R regulates bimodal activation gating of Kv1.2, a novel phenotype among the Kv1.x channel family. While Sig‐1R may not be the only regulator of Kv1.2 bimodal activation gating, our data are a strong indication that the presence of Sig‐1R alone is required for Kv1.2 to occupy the “slow” gating mode.

### Implications of Sig‐1R modulation of Kv1.2 in motor neuron diseases

Native Kv1.x channels often co‐assemble as heterotetramers, and the presence of Kv1.2 has a strong dampening force in the regulation of action potential firing (Palani et al. 2010). The importance of the Kv1.2 subunit in appropriate action potential firing is underscored in Kv1.2 knockout (KCNA2^−/−^) mice, which exhibit seizures and do not survive beyond P19 (Brew et al. 2007; Robbins and Tempel 2012). These mice express heterotetrameric Kv1.x channels that activate at abnormally hyperpolarized membrane potentials, as it has been shown that the threshold for excitability can be altered by adjusting the Kv1.1: Kv1.2 balance toward an increased proportion of Kv1.1 (Brew et al. 2007). Furthermore, bimodal activation gating can be conferred onto the heteromeric Kv1.x channels with only a single Kv1.2 subunit present (Baronas et al. 2015). Thus, Kv1.2 – with its unique ability to display bimodal activation gating – may therefore be disproportionately important among the Kv1.x family in protecting against neuronal hyperexcitability.

As such, Kv1.2 plays an important role in regulating motor neuron hyperexcitability. A recurrent “loss‐of‐function” mutation in KCNA2 was recently identified which results in hereditary spastic paraplegia (Helbig et al. 2016). Hyperexcitability has also been observed in amyotrophic lateral sclerosis (ALS) patients (Nakata et al. 2006; Do‐Ha et al. 2018; Fogarty 2018), with the degree of hyperexcitability correlating with patient survival (Kanai et al. 2012). This may be due to a mechanism involving decreased activity of delayed rectifier potassium channels (Kanai et al. 2006; Do‐Ha et al. 2018). Moreover, reduced delayed rectifier potassium channel current is a mechanism of hyperexcitability in ALS patient‐derived motor neurons (Wainger et al. 2014), and there are reduced expression levels of Kv1.2 in patients with sporadic ALS (Shibuya et al. 2011).

The Sig‐1R is localized to C‐terminals in motor neurons (Mavlyutov et al. 2010), which is disrupted in ALS patients (Prause et al. 2013). Knockout of SIGMAR1 exacerbates ALS in a SOD‐1G93A mouse model by increasing neuronal excitability (Mavlyutov et al. 2013), and disturbing protein and calcium homeostasis in ALS patients (Vollrath et al. 2014). Gene mutations in SIGMAR1 also result in MND (Luty et al. 2010; Al‐Saif et al. 2011; Li et al. 2015; Gregianin et al. 2016; Lee et al. 2016), with the most extensively characterized mutation being Sig‐1R‐E102Q, which results in familial ALS (Al‐Saif et al. 2011). Expression of Sig‐1R‐E102Q results in mitochondrial dysfunction (Tagashira et al. 2014; Fukunaga et al. 2015; Shinoda et al. 2015), ER‐stress‐mediated disruptions in protein homeostasis (Dreser et al. 2017), and inactivation of Kir2.1 potassium channels (Wong et al. 2016).

Our data also reveal that E102 is necessary for Sig‐1R modulation of bimodal activation gating of Kv1.2. How this occurs is unclear as E102 is not part of the ligand binding pocket (Schmidt et al. 2016), but it may play a role in the stabilization of the Sig‐1R following agonist binding. Nonetheless, we predict that the loss of this modulatory interaction disables the ability of Kv1.2 to display acute plasticity and interferes with the adaptability of Kv1.2 to repetitive trains of action potentials. It has been proposed that Sig‐1R‐E102Q‐mediated ALS16 pathogenesis is due to altered ER function and subsequent impaired protein homeostasis (Dreser et al. 2017); however, we suggest that dysfunctional cellular excitability may be upstream of ER‐stress‐mediated pathways. Given that reduced delayed rectifier potassium channel current is a mechanism of hyperexcitability in ALS patient‐derived motor neurons (Wainger et al. 2014) and that hyperexcitability triggers an increase of intracellular calcium leading to ER‐stress‐associated cell death cascades (Pasinelli and Brown 2006; Kiskinis et al. 2014), we propose that dysfunctional regulation of Kv1.2 by Sig‐1R‐E102Q may represent a hitherto uncharacterized mechanism of toxic hyperexcitability in ALS16.

## Conflict of Interest

The authors have no conflict of interest.
